# Effectiveness of a PRECEDE-based education intervention on quality of life in elderly patients with chronic heart failure

**DOI:** 10.1186/s12872-017-0698-8

**Published:** 2017-10-16

**Authors:** Qiong Wang, Lini Dong, Zaijin Jian, Xianghua Tang

**Affiliations:** Department of Geriatrics, The Second Xiangya Hospital, Central South University, Changsha, Hunan 410011 China

**Keywords:** PRECEDE model, Chronic heart failure, Elderly, Quality of life, Self-care behaviors, Depression

## Abstract

**Background:**

One of the most important challenges in public health is to improve the quality of life in patients with chronic heart failure (CHF). Depression, self-care capacity, and quality of life interact each other in these patients. It’s difficult to treat with general education programs and conventional therapy. PRECEDE model is a comprehensive and exclusive theory-based education programs. Its effectiveness for reducing depression and increasing quality of life has been demonstrated in patients with coronary artery bypass grafting, type 2 diabetes, and the elderly. It has not been used in elderly patients with CHF. Thus, this study aims to investigate the effects of this model on self-care behaviors, depression, and quality of life in these patients.

**Methods:**

Patients who met the inclusion criteria were randomly assigned to the intervention or control group. All the patients received conventional medical care. The patients in the intervention group also received 9 sessions of education intervention based on the PRECEDE model and then followed up for 3 months after the intervention. Data were collected before and 3 months after the intervention using 4 questionnaires, namely a PRECEDE-based questionnaire to evaluate predisposing, reinforcing, and enabling factors; the 9-item European Heart Failure Self-care Behavior Scale (EHFScBS-9); the 9-item Personal Health Questionnaire (PHQ-9); and the Minnesota Living with Heart Failure Questionnaire (MLHFQ).

**Results:**

No significant differences were found in the mean scores for the predisposing, enabling, and reinforcing factors, and the mean total scores in EHFScBS-9, PHQ-9, and MLHFQ before the intervention between the intervention and control groups. After the intervention, the scores for the predisposing, reinforcing, and enabling factors increased significantly, and the mean total scores in EHFScBS-9, PHQ-9, and MLHFQ decreased significantly in the intervention group. In addition, these scores significantly differed from those of the control group. Furthermore, the MLHFQ score significantly correlated with the EHFScBS-9 and PHQ-9 scores.

**Conclusion:**

This study demonstrates a trend that PRECEDE model of health education promotion is effective in relieving depression symptoms, enhancing self-monitoring, and improving the quality of life of elderly patients with CHF.

**Trial registration:**

Trial registration number: ChiCTR-IOR-17012779; Trial registry: Chinese Clinical Trial Registry; Date registered: 22 Sep 2017; Retrospectively registered.

## Background

The incidence and prevalence of chronic heart failure (CHF) are increasing because of the increase in the aging population and improved treatment strategies. CHF is a leading cause of morbidity and mortality in the elderly and imposes a heavy economic burden on families and society in general. Patients with CHF are characterized by poor and impaired quality of life because of the unpredictable risk of deterioration and decompensation, coexisting symptoms, high cost of treatment, high readmission rates, and poor prognosis [1]. Individual factors that affect their quality of life include sex, age, New York Heart Association (NYHA) classification, personality, self-care monitoring, knowledge about CHF, education level, financial situation, frequency of hospitalization, co-morbidities, support from family members and caregivers, occupation, and psychological and social factors [2, 3].

Depression is a prevalent comorbidity in patients with CHF, and most patients experience its symptoms. It increases the need for readmission and mortality and has a strong negative impact on the quality of life of patients; a more severe depression is associated with a poorer quality of life [3]. Poor quality of life, in turn, exacerbates the symptoms of depression. Depression is difficult to treat in patients with CHF, as these patients do not benefit from general education programs [4]. A large randomized clinical trial found no significant differences between anti-depression medicines and placebos in reducing depression symptoms or improving cardiovascular status among patients with heart failure and depression [5].

Self-care is an important element in the management of CHF. Accumulating evidence demonstrates that self-care behaviors are important for reducing mortality and morbidity and improving the quality of life. However, inadequate self-care is common in patients with CHF, especially the elderly with a history of depression [6]. Factors that influence the quality of life of patients with CHF also affect the self-care behavior of these patients [7], and the habit of exhibiting poor self-care behavior is difficult to modify [8].

Depression, self-care capacity, and quality of life interact and influence each other. Older age predicts poorer health outcomes and is associated with a more severe depression, greater self-care deficit, and poorer quality of life [3, 6]. Furthermore, inadequate health literacy is especially common in older patients. Thus, to improve the quality of life and outcomes in elderly patients with CHF, comprehensive and exclusive theory-based education programs are required in addition to conventional therapy to target heart failure-related factors.

The PRECEDE planning model used in the present study was developed by Green et al. [9]. PRECEDE stands for predisposing, reinforcing, and enabling constructs in educational/environmental diagnosis and evaluation. The strategy of this model involves thorough analysis of multiple dimensions, including sociology, epidemiology, behavior, environment, education, organization, and management policy, for the development of a health education intervention, implementation of the intervention, and evaluation of the process, impact, and outcomes of the intervention [10]. This model is especially appropriate for application in chronic diseases, and its effectiveness in reducing depression and increasing quality of life has been demonstrated in different subjects, including patients who have undergone coronary artery bypass grafting (CABG) [10], those with type 2 diabetes [11–13], and the elderly [14]. However, to our knowledge, it has not been used in elderly patients with CHF.

Thus, the present study aimed to use the PRECEDE model to optimally tailor a health education promotion program among elderly patients with CHF and investigate its effect on depression, self-care behavior, and quality of life.

## Methods

Between March 2015 and January 2016, patients with CHF who met the inclusion criteria were selected from the Department of Geriatrics and Cardiology of our hospital. Sixty-six patients were recruited and randomly divided into the intervention and control groups in accordance with a randomized computer-generated number table. Four patients were lost to follow-up for various reasons (rate of loss to follow-up, 6.06%). Finally, 31 patients in the control group and 31 in the intervention group completed the study.

The inclusion criteria were CHF with NYHA class II–IV, age ≥ 60 years, presence of typical symptoms, and signs of CHF that disappeared after effective treatment, stable condition, and discharge from the hospital. Patients were excluded when they met the following exclusion criteria: unable to complete the questionnaire; scheduled for an operation within 6 months; had other diseases and an expected survival time < 1 year; had an unstable condition such as that in end-stage heart failure; or had diseases that significantly affected their ability to conduct physical activities.

The general characteristics of the patients were recorded, including name, age, sex, body mass index (BMI), marital and insurance statuses, education level, financial situation, availability of caregivers, comorbidities, time of diagnosis of heart failure, previous hospitalization for heart failure, and NYHA classification.

### PRECEDE model and intervention

Both groups received the same conventional medical care. The intervention group also received 2 months of education intervention based on the PRECEDE model over 9 sessions of 60–90 min each once a week. In the social, epidemiological, and behavioral assessment sessions, social problems and individual factors that had negative effects on the patient’s behavior and quality of life were identified through face-to-face interviews, written materials, lectures, question-and-answer sessions, discussions, and interaction with other patients. These factors can be changed through education. After comprehensive literature reviews and expert consultations, customized educational programs were developed. In accordance with the model, the education promotion program includes the following: 1. Predisposing factors such as motivations for certain behaviors, knowledge about the disease such as the signs and symptoms of decompensation, inducing/aggravating factors, and treatment; familiarization with self-care behaviors; self-care skills; depression and its symptoms; and attitude toward self-care behaviors. 2. Enabling the practice of self-care behaviors, including the acquisition of educational resources such as pamphlets, educational classes, slides, films, and healthcare workers, and instrument support. 3. Reinforcing factors such as rewards and incentives for adhering to the behaviors, including emotional support, recommendation, and encouragement from family members, friends, other patients, and healthcare workers. 4. Practice of self-care behaviors according to the European Heart Failure Self-care Behavior Scale (EHFScBS-9). The program was approved by a panel of senior cardiology experts.

After implementation, the process, impact, and outcome of the intervention were evaluated. Then, the patients were followed up for 3 months. During the follow-up period, the patients continued to participate in the education promotion program and reported their self-care behaviors every 2 weeks.

### Information collection

Four questionnaires were used to collect information. The first was produced based on the PRECEDE model to estimate predisposing (knowledge and attitude), enabling, and reinforcing factors. The knowledge survey comprised 12 items in which the correct answer scored 1 point and a wrong or unknown answer scored 0. The attitude survey for self-care behaviors included 5 items scored on a 5-point Likert scale to indicate the level of agreement, from totally disagree (1 point) to totally agree (5 points). Enabling factors were examined with 6 multiple-choice questions about educational resources, and reinforcing factors were examined with 8 items about the support and encouragement from family, friends, and healthcare workers scored on a 5-point Likert scale.

The EHFScBS-9 questionnaire was used to evaluate self-care behavior. The original scale has 12 items, where each item scores between 1 (completely agree) and 5 points (do not agree at all). The score can range from 12 to 60 points; a lower score indicates better self-care behavior [15]. It has been used in Chinese [16]. In 2009, after analysis of 2592 patients with heart failure from 6 countries, 3 items were deleted and a revised version with 9 items was introduced [17]. The internal consistency and reliability of this revised questionnaire have been confirmed [18]. EHFScBS-9 was used in this study.

The next questionnaire used was the 9-item Patient Health Questionnaire (PHQ-9). It is a self-report tool widely used for screening, diagnosing, and grading depression [19]. The reliability of the Chinese version has also been confirmed [19]. The questionnaire contains 9 items regarding symptoms. The scoring of each item is based on the symptoms of the past 2 weeks, from 0 (“not at all”) to 3 (“nearly every day”), with total scores ranging from 0 to 27. A total score of 0–4 indicates no depression; 5–9, mild depression; 10–14, moderate depression; 15–19, moderate severe depression; and ≥20, severe depression [20]. PHQ-9 has been tested for its reliability and validity in patients with heart failure and can efficiently identify patients with low quality of life [21, 22].

The fourth questionnaire was the Minnesota Living with Heart Failure Questionnaire (MLHFQ), which was used to assess the quality of life in patients with CHF and reliability was validated in Chinese [23]. It has 21 items, which comprise the physical dimension (8 items), emotional dimension (5 items), and additional dimension (8 items). Each item is scored from 0 to 5, and the total score ranges from 0 to 105. A lower score indicates better quality of life [24].

The questionnaires were administered to the patients before and 3 months after the intervention, and information from family members, healthcare workers, and medical records was collected at these time points. All information was collected through face-to-face interviews by a staff who received special training. This study was approved by the institutional ethics committee of the Second Xiangya Hospital of Central South University. All the subjects provided written informed consent.

The SPSS 18.0 software was used for data entry and analysis, and all data were inputted as double entries by two individuals. All data were tested for normal distribution. Data were expressed as mean ± SD. The paired *t* test or Mann-Whitney *U* test was used for comparison between the groups. The Pearson correlation analysis was used to analyze correlations. Differences were considered statistically significant at 2-sided *P* values <0.05.

## Results

No significant differences were found in the mean age of the patients in the intervention and control groups (63.97 ± 14.47 vs. 62.84 ± 13.40, *P* = 0.75). Other general characteristics, including sex, BMI, education level, medical insurance status, the time of CHF onset, NYHA classification, and comorbidities, also showed no significant differences between the groups (*P* > 0.05).

No significant difference was found in the mean scores of the PRECEDE constructs between the intervention and control groups, including predisposing (knowledge and attitude), enabling, and reinforcing factors, before the intervention. However, after the intervention, significant differences were observed between the groups; the mean scores of these factors were significantly increased only in the intervention group (Table 1).

**Table 1:** Mean scores of predisposing, enabling and reinforcing factors in the two groups

Variable	Number of question	Before intervention				After intervention			
		Intervention group	Control group	t	p	Intervention group	Control group	t	p
knowledge	12	2.35 ± 1.43	3.20 ± 1.20	1.637	0.126	9.24 ± 1.03	5.02 ± 1.11	3.121	0.004
attitude	5	6.14 ± 3.10	6.51 ± 2.73	1.030	0.245	20.37 ± 2.16	10.31 ± 3.71	3.937	0.000
enabling factors	6	5.29 ± 4.70	5.08 ± 4.19	0.818	0.678	25.89 ± 3.12	10.97 ± 4.31	4.170	0.000
reinforcing factors	8	10.12 ± 7.80	11.42 ± 9.31	0.975	0.319	34.81 ± 10.95	15.65 ± 9.63	4.763	0.000

Before the intervention, the mean score of each item and the total EHFScBS-9 score were high in both groups, but no significant difference was found between the groups. This suggests that most of the patients lacked self-care behavior, especially about item 1 (I weigh myself every day), which had the highest score. After the intervention, the scores of all items except item 9 (I exercise regularly) and the total scores significantly decreased in the intervention group and were significantly different from those in the control group. This finding showed that the education intervention significantly improved self-care behaviors (Table 2).

**Table 2:** The comparison of EHFScBS-9 scores in the two groups before and after intervention

Items	Before intervention				After intervention			
	Intervention group	Control group	t	p	Intervention group	Control group	t	p
I weigh myself every day	3.90 ± 1.01	4.13 ± 0.72	1.103	0.315	2.03 ± 1.19	3.39 ± 1.02	4.793	0.000
If shortness of breath increases, I contact my doctor or nurse	2.29 ± 1.10	2.55 ± 1.00	0.968	0.337	1.55 ± 0.77	2.23 ± 0.81	3.392	0.001
If legs/feet are more swollen, I contact doctor or nurse	2.65 ± 1.05	2.45 ± 1.06	0.722	0.473	1.61 ± 0.72	2.74 ± 0.89	5.493	0.000
If I gain weight, I contact doctoror nurse	3.90 ± 1.14	3.58 ± 1.03	1.174	0.245	2.03 ± 0.98	3.03 ± 1.08	3.814	0.000
I limit the amount of fluids	3.19 ± 1.28	3.06 ± 1.53	0.418	0.678	2.29 ± 1.04	2.97 ± 1.20	2.380	0.021
If I experience fatigue, I contact doctor or nurse	2.90 ± 1.11	3.19 ± 1.67	1.005	0.319	2.29 ± 0.86	3.06 ± 0.85	3.549	0.001
I eat low-salt diet	2.84 ± 1.00	2.74 ± 0.82	0.417	0.618	1.71 ± 0.59	2.77 ± 1.09	4.797	0.000
I take my medication as prescribed	2.06 ± 1.18	1.90 ± 1.08	0.562	0.576	1.16 ± 0.37	1.87 ± 0.99	3.729	0.000
I exercise regularly	2.65 ± 1.05	2.61 ± 1.20	0.113	0.911	2.19 ± 1.05	2.48 ± 1.09	1.069	0.289
Total scores	26.39 ± 4.81	26.23 ± 4.33	0.139	0.890	16.87 ± 3.34	24.55 ± 3.93	8.282	0.000

No significant differences in the symptoms and the mean total PHQ-9 sore were found between the groups before the intervention. However, after the intervention, some symptoms were significantly relieved, the mean total PHQ-9 score was significantly decreased in the intervention group, and the scores were significantly different between the intervention and control groups (Table 3).

**Table 3:** The comparison of PHQ-9 scores in the two groups before and after intervention

Items	Before intervention				After intervention			
	Intervention group	Control group	t	p	Intervention group	Control group	t	p
Little interest or pleasure in doing things	0.55 ± 1.01	0.94 ± 1.03	1.436	0.156	0.19 ± 0.65	0.87 ± 0.99	3.176	0.002
Feeling down, depressed or hopeless	1.03 ± 1.08	1.06 ± 0.81	0.133	0.895	0.42 ± 0.62	1.03 ± 0.91	3.093	0.003
Trouble falling asleep, staying asleep, or sleeping too much	1.32 ± 1.28	0.77 ± 0.96	1.916	0.060	0.65 ± 1.17	1.00 ± 0.93	1.321	0.191
Feeling tired or having little energy	1.61 ± 0.99	2.03 ± 0.95	1.704	0.094	1.00 ± 1.12	1.42 ± 0.92	1.604	0.114
Poor appetite or overeating	1.55 ± 1.09	1.06 ± 0.53	1.698	0.095	0.39 ± 0.72	1.13 ± 1.09	3.173	0.002
Feeling bad about yourself-or that you’re a failure of have let yourself or your family down	0.23 ± 0.67	0.48 ± 0.85	1.327	0.189	0.06 ± 0.25	0.45 ± 0.72	2.818	0.007
Trouble concentrating on things, such as reading the newspaper and watching TV	0.52 ± 1.09	0.19 ± 0.48	1.508	0.137	0.00 ± 0.00	0.48 ± 0.77	3.503	0.001
Moving or speaking so slowly that other people could have noticed. Or, the opposite-being so fidgety or restless that you have been moving around a lot more than usual	0.32 ± 0.75	0.16 ± 0.45	1.026	0.309	0.00 ± 0.00	0.13 ± 0.43	1.680	0.058
Thoughts that you would be better off dead or of hurting yourself in some way	0.10 ± 0.54	0.11 ± 0.55	0.231	0.818	0.00 ± 0.00	0.42 ± 0.56	4.139	0.000
Mean total score	7.23 ± 5.47	7.03 ± 4.68	0.323	0.747	2.71 ± 2.72	6.94 ± 4.70	4.334	0.000

Before the intervention, the scores for the physical, emotional, and additional dimensions and the total MLHFQ score did not differ significantly between the groups. After the intervention, the individual dimension scores and total MLHFQ scores decreased in both groups, but this was statistically significant only in the intervention group. The differences between the groups were statistically significant (Table 4).

**Table 4:** The comparison of MLHFQ scores in the two groups before and after intervention

Items	Before intervention				After intervention			
	Intervention group	Control group	t	p	Intervention group	Control group	t	p
Physical function	24.39 ± 8.69	23.94 ± 7.35	0.221	0.826	9.13 ± 7.49	13.90 ± 5.59	2.845	0.006
Emotional function	9.10 ± 5.87	9.39 ± 4.74	0.214	0.831	3.48 ± 3.48	7.23 ± 5.84	3.064	0.003
Additional items	21.65 ± 6.92	19.65 ± 6.09	1.208	0.232	8.13 ± 5.88	14.16 ± 4.82	4.418	0.000
Total scores	55.13 ± 16.87	52.97 ± 14.16	0.546	0.587	20.74 ± 14.66	35.29 ± 13.67	4.041	0.000

The Pearson correlation analysis revealed that the MLHFQ score significantly and positively correlated with the EHFScBS-9 (*r* = 0.463, *P* < 0.001) and PHQ-9 scores (*r* = 0.584, P < 0.001) after the intervention. These findings indicate that the symptoms of depression and self-care ability directly influence the quality of life of elderly patients with CHF.

## Discussion

The findings of this study showed that after the education intervention based on the theory of the PRECEDE model, the scores for predisposing, reinforcing, and enabling factors increased significantly and the mean total scores in the EHFScBS-9, PHQ-9, and MLHFQ decreased significantly in the intervention group. Furthermore, these scores differed significantly from the corresponding scores in the control group. The MLHFQ score significantly correlated with the EHFScBS-9 and PHQ-9 scores.

The mean scores of the PRECEDE model constructs, including knowledge, attitude, and enabling and reinforcing factors, increased after the intervention in this study. This finding is consistent with those of previous studies conducted in patients with CABG [25]. Health literacy (predisposing factors) is “the capacity to obtain, read, understand, and process health-related information” [26]. Studies have shown that old age is associated with poor health literacy, which in turn leads to poor outcomes [26, 27]. Social support includes informational (predisposing factors), instrumental (predisposing and enabling factors), and emotional supports (reinforcing factors) [28]. It is associated with depression, self-care, and quality of life, particularly in the emotional dimension, among older patients with CHF [28]. These results indicate that targeting these factors may improve patient outcomes. Previous family or nursing support programs have been shown to improve self-care behaviors and quality of life in patients with heart failure [29, 30]. Our results confirmed these findings and revealed the possibility that the education intervention based on the PRECEDE model focuses on multiple factors and is effective in understanding and adhering to disease management strategies, thereby enhancing self-care behaviors.

Lack of self-care behaviors and presence of depression symptoms are common among patients with CHF, especially the elderly, as shown in our study. The patients had high EHFScBS-9 and PHQ-9 scores before the intervention, which suggests the necessity of an education intervention. Self-care management, depression, and quality of life have reciprocal causation and exacerbate one another. EHFScBS-9 scores have been shown to correlate with depression [7]. Depression leads to a lack of motivation and enthusiasm, and compromises adherence to self-care behaviors. Inadequate self-care behaviors and depression are predictors of poor quality of life and are associated with readmission and high mortality rates [22, 31]. The results of the present study also showed that quality of life positively correlated with the self-care behaviors and symptoms of depression in the elderly patients with CHF.

Other health education strategies have been reported to benefit patients with CHF. For example, the self-management education programs are effective in increasing quality of life and treatment satisfaction [32], and the supportive educational nursing care programs can alleviate fatigue and improve quality of life [30]. Self-care programs can ameliorate heart function and reinforce the patient’s ability and efficiency to perform self-care [33], and home-based disease management programs can improve psychological status, including depression and anxiety [34]. These studies provide evidence that apart from standard therapy, patients with heart failure can additionally benefit from special education promotion programs.

Previous studies confirmed the effectiveness of the PRECEDE model-based education intervention for controlling depression symptoms and severity, and improving sleep status and quality of life in patients with CABG [10, 25, 35]; improving the quality of life of the elderly [14]; and enhancing self-care behaviors and control of HbA1c level, blood pressure, lipid profile, and BMI in patients with type 2 diabetes [11–13]. To our knowledge, its effect in elderly patients with CHF has not been investigated before. The present study expanded the application the PRECEDE model-based education intervention and, for the first time, demonstrated its usefulness in relieving depression symptoms, promoting self-care monitoring, and eventually improving the quality of life of elderly patients with CHF. In the present study, although conventional therapy also improved the patients’ quality of life, the intervention group showed much greater improvements. In addition, after the intervention, no improvements were observed in the score in the “exercise regularly” item of EHFScBS-9, possibly because the subjects in this study were elderly patients, who tend to be frail and have more disabilities and co-morbidities than young adults, which influence their activities.

Although the study demonstrates PRECEDE-based education model is effective in relieving depression symptoms, enhancing self-monitoring and improving the quality of life in elderly patients with CHF, the limitation of our study is the numbers of subjects are small. It would not be enough to draw a meaningful conclusion. Further studies will be needed with a larger patient number.

## Conclusions

Our study demonstrated a trend that the education promotion program based on PRECEDE maybe a useful method to enhance self-care behaviors, improve the depressive state and quality of life in elderly patients with CHF. It provides a rational foundation for future large-scale clinical trials that could further demonstrate the application and effectiveness of this education model.

## Abbreviations

BMI: Body mass index
CABG: Coronary artery bypass grafting
CHF: Chronic heart failure
EHFScBS-9: 9-item European Heart Failure Self-care Behavior Scale
MLHFQ: Minnesota Living with Heart Failure Questionnaire
NYHA: New York Heart Association
PHQ-9: 9-item Personal Health Questionnaire
